# Quantitative Metastatic Lymph Node Regions on Magnetic Resonance Imaging Are Superior to AJCC N Classification for the Prognosis of Nasopharyngeal Carcinoma

**DOI:** 10.1155/2018/9172585

**Published:** 2018-12-02

**Authors:** Xin Zhou, Xiaomin Ou, Youqi Yang, Tingting Xu, Chunying Shen, Jianhui Ding, Chaosu Hu

**Affiliations:** ^1^Department of Radiation Oncology, Fudan University Shanghai Cancer Center, Shanghai 200032, China; ^2^Department of Oncology, Shanghai Medical College, Fudan University, Shanghai 200032, China; ^3^Department of Diagnostic Radiology, Fudan University Shanghai Cancer Center, Shanghai 200032, China

## Abstract

**Purpose:**

Quantitative lymph node burden has been demonstrated to be a critical prognosticator in various malignancies, yet it was seldom explored in nasopharyngeal carcinoma (NPC). This study aimed to investigate the impact of the number of metastatic lymph node regions (LRN) on prognosis of NPC and to establish a new N classification system based on LRN.

**Methods and Materials:**

The magnetic resonance images (MRI) of 354 nondisseminated NPC patients before radical treatment were retrospectively evaluated. The regions with positive lymph nodes (LNs) were quantified according to 2013 updated guidelines for neck node levels. Prognostic value of LRN on distant metastasis-free survival (DMFS) was analyzed using multivariable Cox model after adjusting for other nodal characteristics and therapeutic factors.

**Results:**

LRN strongly correlated with the size, laterality, level, extracapsular extension (ECE), and necrosis of LNs. Risk of distant metastasis significantly escalated with increased LRN (P<0.001). 5-year DMFS for LRN 0-1, 2-6, and *⩾*7 was 97.0%, 86.7%, and 69.7%, respectively. In multivariable Cox analysis, LRN (HR 2.45; 95% CI 1.55-3.88; P<0.001) and maximal LN diameter (MLD) >6cm (HR 4.11; 95% CI 2.23-7.56; P<0.001) were identified as independent predictors of DMFS. Laterality and level showed no prognostic significance when accounting for LRN. A novel N classification scheme was derived by recursive partitioning analysis based on LRN and MLD. Compared with the 7th and 8th edition of American Joint Committee on Cancer (AJCC) systems, the new stratification exhibited better accuracy in predicting survivals.

**Conclusions:**

LRN is a promising quantitative predictor of survival in NPC, eclipsing other classic LN characteristics in prognostic value. The simplified N classification scheme with LRN and MLD is predictive and practical, thus warranting further validation in future.

## 1. Introduction

Nasopharyngeal carcinoma (NPC) is one of the most common head and neck cancers in China and Southeast-Asia. The last decade has seen significant improvements on the loco-regional control rate of NPC owing to the advances in treatment modality and techniques. However, distant metastasis (DM) remains common and has become the major cause of mortality for NPC [1]. Prediction and risk stratification of distant metastasis prior to treatment are critical for therapeutic decision. The N classification in the current tumor, node, metastasis (TNM) staging systems is one of the most important predictors of DM for nondisseminated NPC. However, even the latest 8th edition of N classification by American Joint Committee on Cancer (AJCC) has limitations when applied to different groups of patients. For instance, although Pan et al. and Tang et al. reported good discrimination capacity with the 8th N classification [2, 3], it failed to separate the overall survival (OS) between N2 and N1, or N3 and N2 in another two cohorts [4, 5]. Therefore, further improvement on system robustness is still needed.

The current N classification system does have limitations. Firstly, based on two-categorical nodal laterality, level, and size, the N classification may miss the importance of quantitative lymph node (LN) burden; for instance, patients with extensive metastatic LNs could be staged the same as those with single LN, yet they empirically fare much poorer prognosis. Secondly, the use of multiple parameters may bring more confounders and increase the interobserver inconsistency of N classification. Meanwhile, the use of two-category variables may cause vital loss of information. In addition, the current N classification system was derived from source datasets with OS as endpoint, which was confounded by deaths from local recurrence (T) and thus was unable to distinguish the specific pattern of failure related to nodal metastasis; distant metastasis-free survival (DMFS) would be a more reasonable endpoint to distinguish the actual effect of N on prognostication.

The number of metastatic LNs is a promising novel predictor of survival with demonstrated superiority to the 8th edition AJCC N classification in a variety of squamous cell head and neck cancers [6–9]. As a quantitative variable, it was believed to better reflect the metastatic LN burden and yield superior prognostication efficiency and thus was incorporated into the N classification in a variety of malignancies. However, this effect has never been investigated in nasopharyngeal carcinoma. In NPC, pathological quantification of LNs is unavailable, given the fact that radiotherapy and chemotherapy, rather than surgery, are the mainstay of treatment modality. However, the 2013 consensus guideline for definition of cervical node levels [10] provides a possibility to evaluate the number of metastatic LN regions (LRN) on magnetic resonance imaging (MRI). Therefore, in this study, with the hypothesis that pretreatment quantitative LRN can serve as an indicator of metastatic nodal burden and independent prognostic factor in NPC, we retrospectively investigated the impact of LRN on DMFS and sought to establish a simplified N classification schema with reduced variables.

## 2. Methods and Materials

### 2.1. Patient Population

A total of 354 consecutive nonmetastatic NPC patients treated at our center between September 2010 and March 2011 were included in this study. Each patient underwent a pretreatment workup of complete physical examination, laboratory tests, endoscopy, MR imaging of head and neck, positron emission, and computer tomography (PET/CT) or a combination of chest computed tomography (CT), abdominal sonography/CT, and bone scintigraphy to exclude distant metastases. Those with previous history of cancers or incomplete MR images were excluded.

Medical records and MR images were retrospectively reviewed for this study under approval of the Institutional Review Board. Patients were then restaged using the 8th edition of AJCC staging system.

### 2.2. Image Assessment

All MR images were acquired on a standard 1.5-Tesla scanner using a head and neck coil. Based on the T1-weighted fast spin-echo (FSE) images in axial and sagittal planes, T2-weighted FSE images in the axial plane, as well as postcontrast T1-weighted images with fat saturation in axial and coronal planes, radiological features were reassessed by an experienced radiologist and a radiation oncologist independently. At their disagreements, discussion was necessary to make a consensus.

Diagnostic criteria for retropharyngeal lymph node (RPN) metastasis include (1) lateral RPN with minimal axial diameter (MID)≥5mm; (2) grouping RPNs; (3) RPNs of any size with central necrosis; (4) any visible RPNs in the medial group. Criteria for metastatic cervical lymphadenopathy include (1) MID≥10mm for individual LNs; (2) borderline MID of 8-10mm for three or more contiguous LNs; (3) nodes of any size with central necrosis or extracapsular extension (ECE) [11–13]. Central necrosis was diagnosed in the presence of inhomogeneous signal intensity in LNs (typically high on T2-weighted and low on T1-weighted images) and hypointense nonenhancing areas on postcontrast images; ECE was defined as indistinct nodal margins, nodal capsular enhancement, or infiltration of surrounding fat or muscle planes [14] or fusion with adjacent LNs. Maximal LN diameter (MLD) was defined as the greatest size measured in axial, sagittal, or coronal planes.

The distribution of lymph nodes was mapped following the updated 2013 consensus guideline of node level delineation for head and neck tumors [10]. Assessed subregions included bilateral Ia, Ib, IIa, IIb, III, IVa, IVb, Va, Vb, Vc, VIa, VIb, VIIa, VIIb, VIII, IX, Xa, and Xb. Lower neck involvement was defined as LN metastasis to subcricoid regions including IVa-b and Vb-c. Supraclavicular fossa (SCF) metastasis was defined as involvement of level IVb (medial SCF group) or Vc (lateral SCF group). When calculating LRN, bilateral retropharyngeal space was considered as one unit. LNs located in the border of neighboring levels were recorded as involving both regions.

### 2.3. Treatment

All patients received definitive intensity modulated radiation therapy with simultaneous integrated boost technique (SIB-IMRT), with a prescribed dose of 66-70.4 Gy in 30-32 fractions to primary tumor, 66 Gy to metastatic cervical nodes, 60 Gy to high-risk subclinical and nodal regions, and 54 Gy to elective low-risk nodal regions. All target volumes were delineated according to the definition of International Commission on Radiation Units and Measurements Reports 50, 62, 71, and 83. Residual disease was treated with dose boost using external beam IMRT, or brachytherapy to local residue and electron beam irradiation to palpable nodes. Details of our institutional radiation protocol have been previously reported [15].

Most patients with locoregionally advanced NPC (stages III–IVB in 7th edition of AJCC system) and part of stage II cases with bulky nodes were given cisplatin-based concurrent chemotherapy with/without neoadjuvant/adjuvant chemotherapy, while early stage patients (T1-2N0) received radiation only. Neoadjuvant/adjuvant chemotherapy regimens included 2-3 cycles of alternative docetaxel/cisplatin/fluorouracil (TPF), docetaxel/cisplatin (TP), cisplatin/fluorouracil (PF), and gemcitabine/cisplatin (GP). Concurrent cisplatin was administered weekly or every 3 weeks.

### 2.4. Follow-Up

Follow-up frequency of patients was every 3 months for the first two years and then every 6 months thereafter. MRI of head and neck was performed every 3-6 months. Chest CT, abdominal sonography/CT were done at least annually. Bone scintigraphy or PET/CT was recommended at the discretion of doctors when there are patient-reported new symptoms. Close follow-up tests were suggested at the existence of suspected radiologic findings. Follow-up duration was measured from the date of histological diagnosis, and endpoints of interest included DMFS-time to distant metastasis, OS-time to death of any cause, and disease-free survival (DFS)-time to recurrence or death of any cause.

Suspected distant recurrence was based on (1) appearance of new isolated or multiple lesions in remote regions, including distant lymph nodes, lung, bone, liver, or others, as detected by PET/CT or conventional work-up of surveillance; (2) a progressive change in size/number of lesions within a period of close follow-up; (3) typical radiological characteristics identified under the consensus of at least two radiologists; (4) no evidence of a second primary tumor. Verification of distant metastasis was based on image-guided biopsy or surgical histology when indicated.

### 2.5. Statistical Analysis

Baseline nodal characteristics in patients with different LRN were compared with Chi-square test. Actuarial survival rates were estimated by the Kaplan-Meier method and compared with the log-rank test. Maximally selected rank statistics, as described by Lausen et al. [16], were used to identify the optimal cut-off points for LRN subgroups (R, maxstat package). Univariable Cox regression analysis was performed to assess the association of clinical factors with DMFS, followed by a multivariable stepwise Cox proportional hazards model for confounder adjustment. A new N classification strategy was devised via recursive partitioning analysis (RPA) by including all independent predictors of DMFS. Harrell's concordance index (c-index) and Akaike information criterion (AIC) were used to compare the performance of new N classification with the 7th and 8th edition of AJCC staging system. All statistical analyses were performed using Statistical Product and Service Solutions 19.0 (SPSS, Chicago, IL, USA) and R package (Version 3.3.3, http://www.R-project.org). A two-sided P value of <0.05 was considered statistically significant.

## 3. Results

Demographic and clinical characteristics of the included 354 patients are listed in Table 1. More than 70% of the patients were locoregionally advanced cases, 84.7% underwent chemotherapy, and 34.5% received a cumulative cisplatin dose of over 300mg/m^2^, a cut-off value identified as independent predictor of survival in our previous study on 869 NPC patients receiving IMRT [15].

With a median follow-up duration of 63 months, the actuarial 5-year OS, DMFS, and DFS were 84.4%, 85.0%, and 73.7%, respectively. 5-year DMFS was 96.4% (N0), 92.5% (N1), 86.0% (N2), 45.2% (N3a), and 57.8% (N3b), respectively according to the 7th edition of N classification and 96.4% (N0), 92.3% (N1), 81.5% (N2), and 70.3% (N3) to the 8th edition. 5-year DFS was 90.8%, 83.9%, 69.1%, 45.2%, and 45.9% according to the 7th edition and 90.8%, 83.3%, 65.4%, and 59.5% to the 8th edition. 5-year OS was 94.1%, 89.9%, 84.5%, 61.5%, and 67.6% according to the 7th edition and 94.1%, 89.6%, 80.6%, and 77.3% to the 8th edition.

In this study, two cut points for LRN were identified for risk stratification using maximally selected rank statistics. Patients with 0-1 LRN were found with the lowest risk of DMFS, and those with more than 7 LRN had the highest risk. Therefore, LRN was stratified into three categories: LRN 0-1, LRN 2-6, and LRN*⩾*7.

### 3.1. Correlation between LRN and Other Nodal Features

The frequency of LN involvement in each level was Ib 5.1%, IIa 58.5%, IIb 76.8%, III 56.7%, IVa 19.6%, IVb 8.6%, Va 30.5%, Vb 11.5%, Vc 2.6%, VIIa 76.6%, VIIb 2.3%, and VIII 4.4%. No metastasis to level Ia, VI, IX, or X was found. The median count of LRN was 4 (range 0-20). As a continuous variable, LRN was statistically correlated with other nodal characteristics, including N classification, LN laterality, level, size, level, ECE, and necrosis (P<0.001). The correlation between categorical LRN and other LN features is shown in Table 2. With increment of LRN, there was significantly higher percentage of SCF/lower neck metastasis, > 6cm LNs, and bilateral LNs, as well as ECE/necrosis of LNs. When compared with the 8th AJCC N classification, the concordance of LRN groups with N0-1, N2, and N3 was 44.1% (78/177), 63.2% (55/87), and 71.1% (64/90), respectively.

### 3.2. Impact of Nodal Variables on Survival

**Figure 1:**
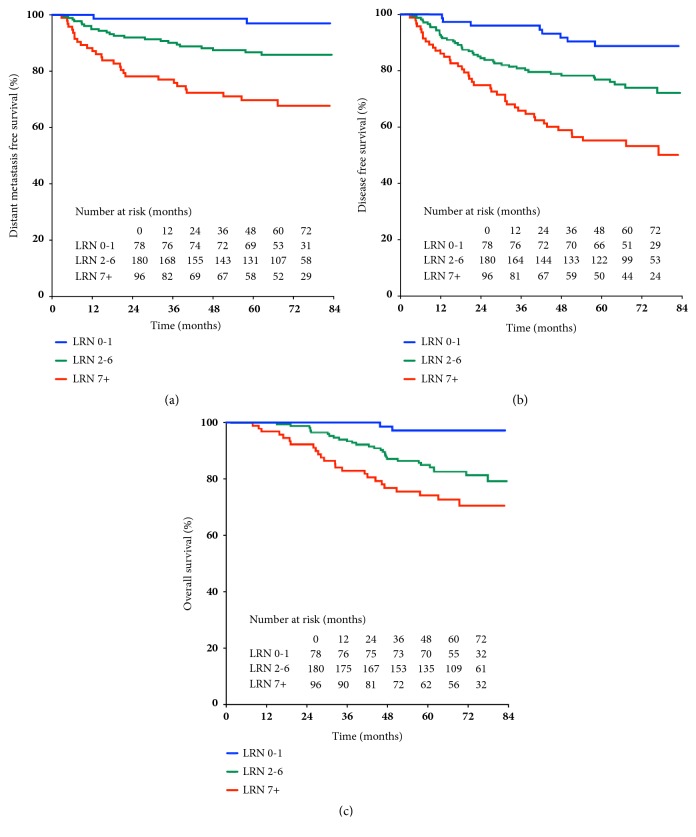
Kaplan-Meier estimate with the three-categorical LRN on (a) distant metastasis-free survival; (b) disease-free survival; (c) overall survival. LRN: number of metastatic lymph node regions.

DMFS was chosen as primary endpoint in our analysis based on the following considerations: (1) distant metastasis has become the major pattern of failure for NPC nowadays; (2) the commonly used endpoints, OS and DFS, could easily be complicated by local failure (T factor), making it difficult to distinguish the actual effect of N factor on survival; (3) LN metastasis has been well known with its impact on distant dissemination. According to univariable analysis in Kaplan-Meier method, LRN, whether as a continuous or categorical variable, was strongly predictive for worsening DMFS (P<0.001). When using the three-categorization stratification, the estimated 5-year DMFS for LRN 0-1, 2-6, and ≥7 was 97.0%, 86.7%, and 69.7%, respectively; 5-year DFS was 88.7%, 76.9%, and 55.2%, respectively; 5-year OS was 97.1%, 84.9%, and 74.2%, respectively (Figure 1). Other significant factors for DMFS included the 8th edition of AJCC T and N classification and all the other nodal characteristics; as a therapeutic prognosticator, cumulative cisplatin dose*⩾*300mg/m^2^ improved 5-year DMFS from 80.5% to 89.6% (P=0.040) (Table 3).

By including these significant factors in a stepwise backward selection procedure, multivariable Cox regression model found LRN, MLD> 6cm, and cumulative cisplatin dose as independent predictors for DMFS (P<0.05), while ECE retained a marginal significance (P=0.08) (Table 3). T and N classification by 8th edition of AJCC system, nodal level, laterality, and necrosis were excluded as insignificant factors by the multivariable model.

### 3.3. A Novel N Classification Schema Based on LRN

**Figure 2:**
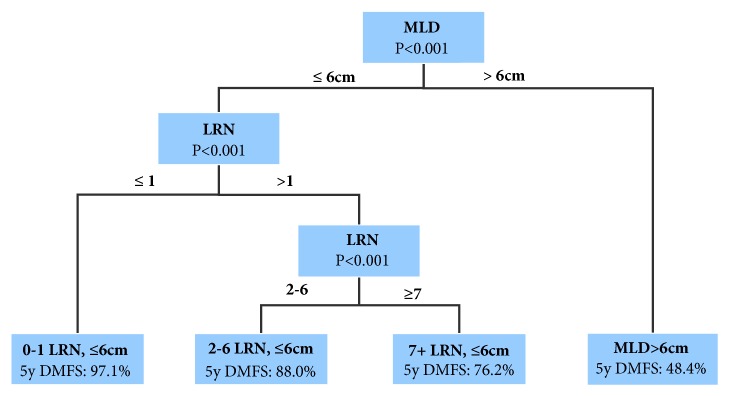
Proposed N classification derived from recursive partitioning analysis in patients with nasopharyngeal carcinoma. MLD: maximal lymph node diameter; LRN: number of metastatic lymph node regions; DMFS: distant metastasis-free survival.

As pretreatment nodal features, LRN and MLD were chosen into recursive partitioning analysis for clustering of DMFS risk. ECE dropped out of the model relative to other covariables. The conditional inference tree was plotted as in Figure 2. Hence a new N stratification schema was generated as follows: Group 1 (22.0%), LRN 0~1 and MLD⩽6cm; Group 2 (47.4%), LRN 2~6 and MLD⩽6cm; Group 3 (21.8%), LRN 7+ and MLD⩽6cm; Group 4 (8.5%), any LRN, MLD>6cm. The estimated 5-year survival of Groups 1-4 was DMFS, 97.1%, 88.0%, 76.2%, and 48.4%; OS, 97.1%, 86.6%, 77.1%, and 61.0%; DFS, 88.7%, 79.0%, 59.7%, and 41.5% (all P<0.001).

**Figure 3:**
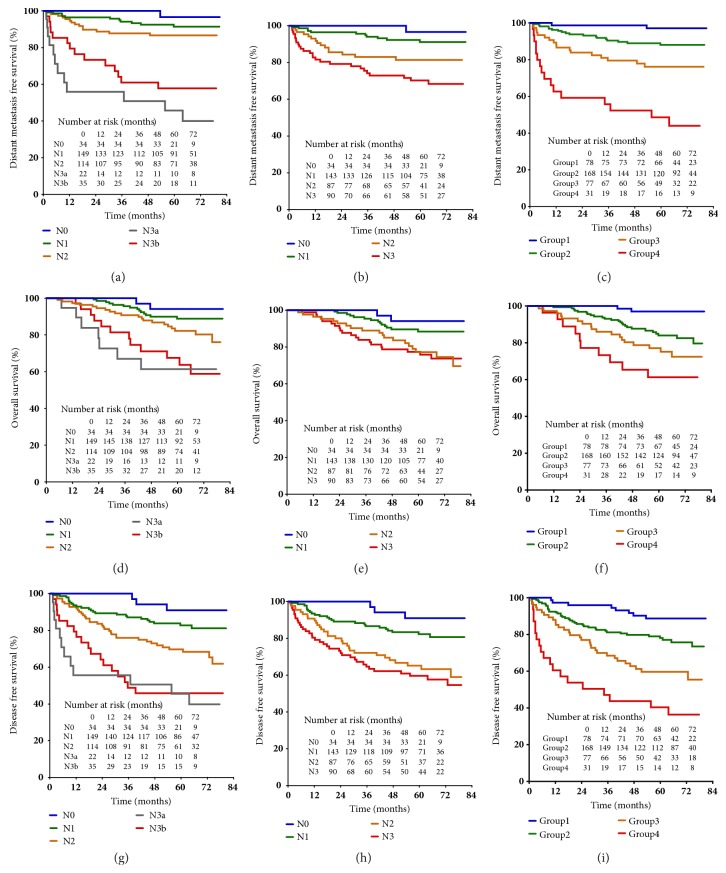
Kaplan-Meier estimate with the 7th edition (left), 8th edition (middle), and the proposed N classification (right) on (a–c) distant metastasis-free survival, (d–f) overall survival, and (g–i) disease-free survival.

Compared with the 7th and 8th edition of AJCC N classification, the new N classification showed improved discrimination capability of DMFS (c-index 0.74, 0.69 in 8th, 0.72 in 7th), OS (c-index 0.71, 0.66, 0.68), and DFS (c-index 0.70, 0.67, 0.69), while information loss of the model was reduced (AIC for DMFS 2234, 2242, and 2235; AIC for OS 2151, 2157, and 2154; AIC for DFS 2261, 2267, and 2262). Kaplan-Meier estimates of survival by the three systems were shown in Figure 3.

## 4. Discussion

Over the past decades, a cumulating body of data has highlighted the prognostic importance of quantitative LN burden for malignant tumors. In gastric cancer [17], breast cancer [18], and colorectal cancer [19], the number of metastatic LNs has been reported to profoundly correlate with overall survival and hence was incorporated into the AJCC staging system. For head and neck squamous cell carcinoma, recent evidence showed that the number of LNs on postsurgical pathology is an independent predictor of mortality in oral cavity cancer [6], oropharynx cancer [7], and hypopharyngeal and laryngeal cancer [8], with a prognostic accuracy outweighing LN size, laterality, and even the overall N classification by current AJCC systems. Similar impact has been found in papillary thyroid cancer, where risk of OS in young patients could be further stratified on the basis of metastatic node number [9]. These findings indicated that quantitative metastatic LN burden may have a universal prognostic value for different malignancies.

However, such effect has never been investigated in NPC, primarily due to the difficulty of LN quantification in a nonsurgical setting with no histological evaluation. Moreover, as ECE is not rare in NPC [20], multiple bulky LNs fused as one would increase the uncertainty of LN numeration on radiological images. In this study, we adopted LRN as a surrogate marker to represent the extent of LN metastasis, based on the definition of LN levels in the 2013 guideline. The procedure of LRN quantification was easy to perform without causing much extra burden to clinical work.

To our knowledge, this is the first study to demonstrate the prognostic value of quantitative LN regions in NPC patients. We identified two cut-off points of LRN to generate a three-category stratification. It turned out that LRN was significantly correlated with other nodal features, indicating that anatomical spread of LNs is usually accompanied with ECE, necrosis, and enlargement of LNs. Survival analysis suggested that incremental LRN strongly correlated with increased DM risk. After adjusting for therapeutic factors and potential confounders, LRN remained a predominant predictor for DMFS. The underlying mechanism might be that higher LRN reflects increased tumor burden, which is highly associated with distant metastasis and overall survival [21]. On the other hand, massively spread-out LNs might denote the biological aggressiveness of cancer clones driven by factors such as lymphangiogenesis [22], which facilitates systemic dissemination in NPC.

Our data also confirmed the prognostic value of LN size in NPC, which remained controversial in previous reports. According to Lee et al. [23] and Heng et al. [24], MLD on palpation was an independent predictor of survival in NPC. However, subsequent studies by Mao et al. [20] and Li et al. [25] measured LN size on MRI and found that maximal axial diameter (MAD), whether as multicategorical variable or with a cut-off value of 3cm, failed to reach any statistical significance on prognosis. These studies, though, should be interpreted with caution, because axial measurement can not depict the panorama of LNs. In fact, LN size might be remarkably underestimated in these studies (only 6/924 with MAD>5cm, by Mao; 1/749 with MAD>6cm, by Li). In our data, 8.8% (31/354) patients had LNs with MLD>6cm, 96.8% (30/31) of which were acquired in the coronal MRI plane, suggesting that large LNs are not rare on three-dimensional projection, and axial plane is definitely not enough for LN measurement. In comparison, by introducing detailed three-dimensional measurement data, our study identified MLD as an independent prognostic factor for DMFS (HR 4.11, 95% CI 2.23-7.56); addition of MLD>6cm further refined the risk stratification on the basis of LRN. Our study supports the prior findings by Lee and the inclusion of LN size in the 8th edition of AJCC N classification system. Given the lack of three-dimensional radiological data of LN size in previous reports, we strongly recommend this information be included in subsequent studies, so as to further elucidate the prognostic impact of MLD in NPC.

A related finding of the present study is that when accounting for LRN, classic variables in AJCC N classification including LN laterality, SCF level, and lower neck level were no longer significant prognostic factors for DMFS. Correlation analysis showed strong collinearity between all these variables and LRN, suggesting that they might be surrogates for quantitative LN burden. This was in concordance with previous evidence in other head and neck cancers, where features like LN contralaterality were eclipsed by LN number in prognostic value [6, 26].

In NPC, removal of lower neck and SCF from the prognostic model was unexpected but could be properly explained by the latest advances in LN biology. Emphasis on the importance of SCF over long time was based on its proximity to the thoracic duct, which possibly mediates systemic dissemination via lymph-venous conjunction [27]. However, this concept is being questioned by growing evidence in clinical and basic scientific researches. Recently, two fundamental researches revealed that DM is driven by direct invasion of tumor cells into LN blood vessels [28] rather than through the thoracic duct [29] and that early DM can happen without relying on sequential lymphatic drainage. This echoes the emerging fact that intensified treatment of locoregional disease failed to translate into benefit of DMFS in NPC. Substantially, LN metastasis to lower neck or SCF might act merely as indicators of higher potential of DM in NPC, rather than as direct precursors. However, it would be reckless to abolish the importance of SCF simply with results of this study, and the actual biological role of SCF in distant metastasis for NPC remains to be uncovered.

We proposed a novel N classification schema using recursive partitioning analysis algorithm. By retaining LRN and MLD for risk stratification, the new system was simplified, showing an improved predictive power of survival over the AJCC (7th and 8th edition) staging systems. The potential advantages of the new schema include the following: (1) it is based on LRN, an independent factor that drives outcomes, rather than surrogates; (2) the three-categorization criteria of LRN better partitioned risk than the classic two-category criteria did, with reduced information loss; (3) 3-dimensional measurement of MLD was more reasonable for outlining the prognostic value of LN size. Our system appeared also superior to the historical reports on 8th staging, with a higher c-index in both DMFS and DFS [2, 3]. Collectively, the proposed N classification may offer new direction for selecting NPC candidates that benefit from more intensified systemic treatment, such as induction/adjuvant chemotherapy and high-dose concurrent cisplatin.

In our patient population, the 8th edition of AJCC N classification did not show superiority to the 7th edition in prognostic power, especially in distinguishing OS of N2 and N3 (P>0.05). This was quite similar to Yang's reports, which failed in separating N1 with N0, and N3 with N2 by using the 8th edition [5]. A plausible reason is that replacing SCF with lower neck led to a higher percentage of upstaging from N1-2 to N3 (9.3%) in our study, possibly diluting the distinction between N3 and N2. Therefore, the discrimination capability of the 8th N classification may subject to the composition of patients. This internal deficiency of robustness remains to be further discussed in future series.

The present study had some limitations. Being conducted in an institutional population, it might require external validation with larger cohorts in future. Besides, the conclusions are confined by the retrospective nature of this study. In addition, use of PET/CT was limited in this study due to the problem of reimbursement in China. More data of PET/CT guided LN evaluation should be incorporated in future, according to the recommendation of National Cancer Comprehensive Network (NCCN) guideline. Moreover, well-known prognostic factors such as tumor volume and Epstein-Barr virus DNA were not included in this study; it is yet unknown if incorporating these factors will alter the conclusions of this study. Further efforts of incorporating these predictors into the current scheme will be worthwhile.

## 5. Conclusions

In summary, our study demonstrated that LRN is an independent predictor of DMFS in patients with NPC, predominantly outweighing other classic factors such as LN laterality and level in prognostic value. By combining LRN and MLD, the novel N classification confers significant improvement over the present staging systems in prognostication. Future data for validation of this schema will be warranted.

## Figures and Tables

**Table 1:** Patient characteristics.

Characteristics	No. of patients (%)

Age, median (range), y	49 (12-81)

Gender	
Male	266 (75.1)
Female	88 (24.9)
Histological type	
WHO I	1 (0.3)
WHO II	353 (99.7)
T classification (AJCC 7^th^/8^th^)	
T1	119 (33.6)/119 (33.6)
T2	52 (14.7)/64 (18.1)
T3	47 (13.3)/88 (24.9)
T4	136 (38.4)/83 (23.4)
N classification (AJCC 7^th^/8^th^)	
N0	34 (9.6)/34 (9.6)
N1	149 (42.1)/143 (40.4)
N2	114 (32.2)/87 (24.6)
N3	57 (16.1)/90 (25.4)
Clinical stage (AJCC 7^th^/8^th^)	
I-II	100 (28.3)/102 (28.8)
III-IV	254 (71.7)/259 (71.2)
Treatment modality	
RT alone	54 (15.2)
IC+RT	37 (10.4)
CCRT	49 (13.8)
IC+CCRT	138 (38.9)
CCRT+AC	14 (3.9)
IC+RT+AC	62 (17.5)
Cumulative cisplatin dose	
≤ 300mg/m^2^	301 (84.8)
> 300mg/m^2^	53 (14.9)

Abbreviations: AJCC: American Joint Committee on Cancer; RT: radiation therapy; IC: induction chemotherapy; CCRT: concurrent chemoradiotherapy; AC: adjuvant chemotherapy.

**Table 2:** Correlation between LRN and other nodal characteristics.

Variable	LRN			*P*
	0-1	2~6	≥7	
N classification (AJCC 8^th^)				<0.001
N0-1	78 (100%)	99 (55.0%)	0	
N2	0	55 (30.6%)	32 (33.3%)	
N3	0	26 (14.4%)	64 (66.7%)	
SCF involvement				<0.001
SCF (-)	78 (100%)	176 (97.8%)	65 (67.7%)	
SCF (+)	0	4 (2.2%)	31 (32.3%)	
Lower neck involvement				
Lower neck (-)	78 (100%)	162 (90.0%)	35(36.5%)	
Lower neck (+)	0	18 (10.0%)	61 (63.5%)	
Laterality				<0.001
Nil	65 (83.3%)	2 (1.1%)	0	
Unilateral	13 (16.7)	112 (62.2%)	7 (7.3%)	
Bilateral	0	66 (36.7%)	89 (92.7%)	
MLD				<0.001
≤ 6cm	78 (100%)	168 (93.3%)	77 (80.2%)	
> 6cm	0	12 (6.7%)	19 (19.8%)	
ECE				<0.001
Negative	75 (96.2%)	85 (47.2%)	17 (17.7%)	
Positive	3 (3.8%)	95 (52.8%)	79 (82.3%)	
Nodal necrosis				<0.001
Negative	74 (94.9%)	104 (57.8%)	33(34.4%)	
Positive	4 (5.1%)	76 (42.2%)	63 (65.6%)	

Abbreviations: LRN: number of involved lymph node regions; AJCC: American Joint Committee on Cancer; MLD: maximal lymph node diameter; SCF: supraclavicular fossa; ECE: extracapsular extension.

**Table 3:** Univariable and multivariable analyses for distant metastasis-free survival.

Variable		Univariable		Multivariable	
	No. (%)	Hazard Ratio (95% CI)	*P*	Hazard Ratio (95% CI)	*P*
Age			0.118		
≤ 49	177 (50.0)	1 (reference)			
> 49	177 (50.0)	1.02 (0.99-1.04)			
Gender			0.819		
Male	266 (75.1)	1 (reference)			
Female	88 (24.9)	1.07 (0.58-1.98)			
T classification (AJCC 8^th^)			0.019^∗^		
T1-2	183 (51.7)	1 (reference)			
T3-4	171 (48.3)	2.73 (1.34-5.60)			
N classification (AJCC 8^th^)			<0.001^∗^		
N0-1	177 (50.0)	1 (reference)			
N2	87 (24.6)	2.76 (1.46-5.22)	0.009		
N3	90 (25.4)	4.93 (2.78-8.75)	<0.001		
Laterality			0.008		
Nil/Unilateral	199 (56.2)	1 (reference)			
Bilateral	155 (43.8)	2.11(1.22-3.66)			
Lower neck involvement			0.001		
No	275 (77.7)	1 (reference)			
Yes	79 (22.3)	2.49 (1.45-4.36)			
SCF involvement			0.001		
No	319 (90.1)	1 (reference)			
Yes	35 (9.9)	3.72 (2.02-6.86)			
Necrosis					
No	211 (59.6)	1 (reference)			
Yes	143 (40.4)	3.55 (2.00-6.32)			
ECE			0.008		0.080
No	177 (50.0)	1 (reference)		1 (reference)	
Yes	177 (50.0)	3.73 (1.96-7.09)		1.98 (0.92-4.26)	
MLD			<0.001		<0.001
≤ 6cm	323 (91.2)	1 (reference)		1 (reference)	
> 6cm	31 (8.8)	6.20 (3.44-11.14)		4.11(2.23-7.56)	
LRN			<0.001^∗^		<0.001^∗^
0~1	78 (22.0)	1 (reference)		1 (reference)	
2~6	180 (50.8)	5.34 (1.58-17.95)	0.034	4.59 (1.36-15.49)	0.039
≥7	96 (27.1)	13.78 (4.13-45.93)	<0.001	9.78 (2.88-33.25)	0.002
Cumulative cisplatin dose			0.040		0.028
< 300mg/m^2^	301 (84.8)	1 (reference)		1 (reference)	
≥ 300mg/m^2^	53 (14.9)	0.34 (0.12-0.95)		0.32 (0.12-0.88)	

Abbreviations: AJCC: American Joint Committee on Cancer; MLD: maximal lymph node diameter; SCF: supraclavicular fossa; ECE: extracapsular extension; LRN: number of involved lymph node regions.

^∗^Overall P value for multiple categorical variables.

## Data Availability

The original Excel data used to support the findings of this study have not been made available because it is temporarily not allowed according to the institutional regulations.
